# Feasibility of Remote Delivering an Exercise and Lifestyle Education Program for Individuals Living with Prediabetes and Diabetes in Brazil

**DOI:** 10.3390/ijerph192416697

**Published:** 2022-12-12

**Authors:** Mariana Balbi Seixas, Gabriela Lima de Melo Ghisi, Paul Oh, Daniele Sirineu Pereira, Ana Paula Boroni Moreira, Ann Kristine Jansen, Ana Paula Delgado Bomtempo Batalha, Gabriela do Nascimento Cândido, Josiane Aparecida de Almeida, Danielle Aparecida Gomes Pereira, Lilian Pinto da Silva

**Affiliations:** 1 Cardiovascular Research Unit and Exercise Physiology, University Hospital, Federal University of Juiz de Fora, Avenida Eugênio do Nascimento S/N, Juiz de Fora 36038-330, MG, Brazil; 2 Graduate Program in Physical Education, Faculty of Physical Education and Sports, Federal University of Juiz de Fora, Rua José Lourenço Kelmer S/N, Juiz de Fora 36036-900, MG, Brazil; 3 Cardiovascular Rehabilitation and Prevention Program, Toronto Rehabilitation Institute, University Health Network, 347 Rumsey Road, Toronto, ON M4G 1R7, Canada; 4 Department of Physical Therapy, Federal University of Minas Gerais, Avenida Presidente Antônio Carlos, 6627, Pampulha, Belo Horizonte 31270-901, MG, Brazil; 5 Graduate Program in Rehabilitation Sciences, Federal University of Minas Gerais, Avenida Presidente Antônio Carlos, 6627, Pampulha, Belo Horizonte 31270-901, MG, Brazil; 6 Department of Nutrition, Federal University of Juiz de Fora, Juiz de Fora 36036-900, MG, Brazil; 7 Department of Nutrition, Federal University of Minas Gerais, Avenida Presidente Antônio Carlos, 6627, Pampulha, Belo Horizonte 31270-901, MG, Brazil; 8 Graduate Program in Rehabilitation Sciences and Physical-Functional Performance, Faculty of Physical Therapy, Federal University of Juiz de Fora, Avenida Eugênio do Nascimento S/N, Juiz de Fora 36038-330, MG, Brazil

**Keywords:** diabetes mellitus, prediabetes, health education, patient education, exercise training, internet-based intervention, virtual education

## Abstract

This study aimed to test the feasibility of remote delivering a 12-week exercise and lifestyle education program (ExLE) or a 12-week exercise program (Ex) for individuals with prediabetes and diabetes in terms of acceptability, implementation, practicality, and limited efficacy. The programs were internet- or telephone-based delivered, depending on the participants’ internet access and technology literacy. Of the 196 individuals screened, 15 were included in the study (internet-based delivery (*n* = 13); telephone-based delivery (*n* = 2)). Twelve participants completed the program they were randomized to, and most reported being satisfied with the study interventions (acceptability). Data collection procedures, weekly follow-up, study website visits, and educational materials were proper (implementation), and the adherence rate to study interventions ranged from 24% to 58% (practicality). Additionally, both programs (ExLE and Ex) seemed to promote beneficial changes in functional capacity (limited efficacy). The internet-based remote delivery of the interventions showed feasibility. Therefore, in future trials, exercise and educational interventions can be internet-based remote delivered to individuals with prediabetes and diabetes with internet access and technology literacy. In addition, some adjustments to eligibility criteria, study websites, more accessible ways of recording exercise sessions and using educational materials, and an initial supervised exercise session are recommended.

## 1. Introduction

Diabetes is a chronic condition characterized by raised blood glucose levels [1]. Persistently high blood glucose levels cause macro and microvascular damage affecting the eyes, kidneys, nerves and heart [1,2]. In addition, functional capacity is impaired in people living with diabetes [3], and this outcome is related to poor prognosis due to increased cardiac risk and mortality [4,5]. Therefore, diabetes management requires multifactorial and continuous strategies to reduce the risk of acute and chronic complications, including taking medicines, maintaining psychological well-being, and adopting good health behaviors (e.g., smoking cessation, eating a healthy diet, and performing regular exercise) [2,6].

In this context, diabetes education is one of the essential elements of diabetes care [6,7,8], as educational interventions effectively promote behavior changes and can support the self-management of individuals with diabetes, which can ultimately promote metabolic control, as described in previous reviews [9,10,11]. Despite its great potential to support diabetes care, the impact of diabetes patient education on behavior changes has not been frequently evaluated in low- and middle-income countries such as Brazil, with most studies on this topic being carried out in high-income countries [12,13,14].

Technology has been used to deliver diabetes education remotely, mitigating barriers to accessing and expanding the delivery of educational interventions in settings in which health services or patient resources are insufficient or limited [6,15]. In addition, due to the complex scenario of the COVID-19 pandemic and the social distancing measures adopted to prevent the spread and contagion of the virus, widespread attention has recently been paid to the remote delivery of educational interventions [16]. Previous studies have shown the benefits of educational interventions delivered remotely for people living with diabetes and prediabetes, including reduction of glycated hemoglobin (HbA1c) [17,18,19], increase in disease-related knowledge [20], and positive changes in health behaviors [21,22].

The Diabetes College Brazil Study is a randomized clinical trial multicenter designed to assess the effectiveness of an Exercise and Lifestyle Education program (ExLE) on functional capacity, diabetes knowledge, health behaviors, and cardiometabolic health parameters in Brazilians with prediabetes or diabetes (NCT03914924). The Exercise program (Ex) was developed based on the Diabetes Clinical Practice Guidelines [2,6]. The ExLE includes an evidence-based curriculum developed by the University Health Network in Toronto, Canada, named Diabetes College^TM^, which was translated and culturally adapted to meet the educational needs of people living with prediabetes and diabetes in Brazil [23]. Both programs were structured to be delivered in person; however, once the COVID-19 pandemic was declared when the pilot study was underway [24], the research team identified the need to provide the study interventions remotely.

Based on that, this study aimed to evaluate the feasibility of remote delivery of ExLE and Ex programs to individuals with prediabetes or diabetes.

## 2. Materials and Methods

### 2.1. Study Design and Ethical Aspects

This is a multicenter study designed to test the feasibility of remote delivering the ExLE (12-week exercise and educational interventions) and Ex (12-week exercise only) programs for individuals with prediabetes or diabetes. The study took place in two Brazilian cities: Juiz de Fora, Minas Gerais (center 1) and Belo Horizonte, Minas Gerais (center 2). The study protocol was approved by the Research Ethics Committees of the Federal University of Juiz de Fora (UFJF) and the Federal University of Minas Gerais (UFMG), registered on the Open Science Framework platform (https://osf.io/k5yxm/ accessed on 10 December 2022) and reported following the CONSORT checklist for pilot and feasibility trials [25].

### 2.2. Participants

Participants were recruited face-to-face from health services in the two centers and from phone calls addressing a list of participants from previous studies developed by the same research team [26,27,28], which did not involve exercise or educational interventions. In addition, social media advertisements and dissemination of study recruitment by face-to-face and email among local healthcare providers and employees of the UFJF and UFMG were done, respectively. Individuals who met the following criteria were eligible for the study: (C1) clinical diagnosis of prediabetes or diabetes (type 1 or type 2), following the national diabetes guideline [2]; (C2) age ≥ 18 years old; (C3) lack of cognitive limitation (i.e., six-item screener score ≥ 4) [29]; absence of unstable (C4) coronary artery disease, (C5) heart failure, (C6) pacemaker and/or implantable cardioverter defibrillator, (C7) intermittent claudication, (C8) recent history of cardiovascular events or cardiac surgery (≤6 months); (C9) no complex arrhythmias; (C10) no chest pain; (C11) absence of a physical condition that limited or prevented the performance of exercises; (C12) not currently participating in a structured exercise program that follows diabetes guidelines [2,6]; (C13) written physician permission for exercising; (C14) glycemic control (i.e., HbA1c ≤ 7% [30] in a routine blood test performed up to 3 months before the pre-intervention assessment); and (C15) literacy and without reading limitation (e.g., reduced visual acuity).

### 2.3. Procedures

The recruitment lasted six months. The individuals who were eligible for the study and interested in participating received explanations about the study procedures before signing the consent form and were screened for internet access and technology literacy using an instrument developed by the researchers based on best practices in digital health literacy [31]. Based on that screening, the participants were allocated to internet-based (i.e., internet access and technology literate) or telephone-based (i.e., no internet access and/or no technology literate) remote delivery format. Afterward, they were randomized for one of the two programs (ExLE or Ex), totaling four groups: (1) ExLE internet-based; (2) Ex internet-based; (3) ExLE telephone-based; and (4) Ex telephone-based. 

In the pre-intervention assessment, clinical and sociodemographic data were collected. In addition, the heart rhythm was analyzed by resting electrocardiographic monitoring at the CM5 lead, and the study variables were measured. During the 12-week intervention, the research team recorded the resources used and the participants’ questions raised in the weekly follow-up. At intervention completion, the participants’ exercise and study diaries were collected, and the data access registered on the website platform were analyzed. The same variables measured in the pre-intervention assessment were measured in the post-intervention assessment, and the participants answered a satisfaction questionnaire with the interventions.

### 2.4. Study Variables’ Measurement

The pre- and post-intervention assessment took two sequential stages (face-to-face and remote). The following variables were measured in the face-to-face stage: (1) functional capacity measured by the incremental shuttle walking test (ISWT) [32]; (2) physical activity level measured from the average of steps/day and total steps/week collected by a pedometer worn during seven days [33]; (3) anthropometric assessment by body mass index (BMI) and waist circumference measurement; (4) glycated hemoglobin (HbA1c) level obtained from the routine tests dated no more than three months before each study assessment point; and (5) adherence to a Mediterranean food pattern measured by the Mediterranean Diet Scale (MDS) total score [28] (a 13-item instrument with a total score ranging from 0 to 13: higher scores indicate greater adherence to Mediterranean food patterns). The following variables were measured in the remote stage: (6) exercise self-efficacy measured by the self-administered Bandura’s exercise self-efficacy scale (BESES) total score [27] (an 18-item instrument with a total score from 0 to 100: higher scores indicate greater confidence in maintaining an exercise routine); (7) diabetes knowledge measured by the self-administered DiAbeTes Education Questionnaire (DATE-Q) total score [26] (a 20-item questionnaire with a total score from 0 to 20: higher scores indicate greater disease-related knowledge); (8) healthy literacy measured by the Newest Vital Sign (NVS) [34] total score (a 6-item questionnaire with a total score from 0 to 6: higher scores indicate greater health literacy), administered by interview; (9) depression measured by Center for Epidemiological Scale Depression (CESD) [35] total score (a 20-item questionnaire with a total score from 0 to 60: higher scores indicate a higher level of depressive symptoms), administered by interview; and (10) quality of life (QofL) measured by the Medical Outcomes Study 36-Item Short-Form Health Survey total scores of eight health domains: functional capacity, physical aspects, pain, general health status, vitality, social aspects, emotional aspects, and mental health [36] (each domain has a total score from 0 to 100: higher scores indicate better quality of life), administered by interview. In this stage, all the questionnaires were answered remotely under research team monitoring by phone or voice calls via WhatsApp. The researcher administered the ones that should be answered via interview and remained quietly available on the call during the answer to the self-administered ones to clarify points that could arise from the participant. The internet-based groups’ participants received a link by WhatsApp to access the Research Electronic Data Capture (REDCap) [37,38] platform and answered the self-administered questionnaires online. The telephone-based groups’ participants received an envelope containing the printed self-administered questionnaires and answered them on paper.

### 2.5. Interventions

The 12-week exercise intervention consisted of at least 150 min of aerobic exercise per week and 2 to 3 muscle-strengthening sessions per week from the 4th week of the intervention. The exercise sessions were structured in exercise plans as follows: warm-up (stretching exercises and slow walking), aerobic exercise (moderate-to-vigorous intensity walking according to the Borg rating of perceived exertion exercise scale modified [39]), muscle-strengthening exercises (localized muscle endurance exercises that encompass the major muscle groups) and cool down (slow walking and stretching exercises). Participants in the internet-based group accessed weekly exercise plans on the Ex internet-based group website, and only the participants of this group had access to this website link. They also received weekly follow-up text messages via WhatsApp with standardized reminders about exercise goals and the importance of keeping track of their exercise and reporting the exercise sessions in the exercise diary. Participants in the telephone-based group received a printed version of the weekly exercise plans, and the weekly follow-up occurred via phone calls.

In addition to the exercise intervention, participants randomized to the ExLE group received the educational intervention proposed for the Diabetes College Brazil Study, the content of which was previously distributed and organized into 12 weekly lesson plans [23]. A detailed description of educational intervention is available in the patient education program development study published previously [23]. Participants in the internet-based group accessed their weekly lesson plans on the ExLE internet-based group website, and only the participants of this group had access to this website link. The educational content consisted of the following materials: (1) eighteen video lessons (recorded by the research team, lasting approximately 20 min and based on the five pillars of Diabetes College^TM^: treat diabetes, get active, eat healthy, feel well, and take control); (2) twelve videos related to the topics of the week (THRiVE, i.e., videos that integrate principles of chronic disease management and behavior changes to help patients to develop self-management skills through goal setting and action planning); and (3) an online version of the Patient Guide (A Guide to Help You Live and Thrive with Diabetes, containing 298 pages and 17 chapters organized into five sections). In addition, they received weekly WhatsApp text messages from the research team to remind them about the materials in their lesson plan and the importance of keeping track of access to educational content and reporting it in the study diary. Participants in the telephone-based group received a printed version of the weekly lesson plans. The educational content consisted of: (1) the transcription of video lessons; (2) the transcription of videos related to the topics of the week—THRiVE; and (3) a printed version of the Patient Guide. They also received weekly phone calls from the research team to remind them about the materials in the week’s lesson plan and the importance of keeping track of the educational content reading and reporting it in the study diary.

### 2.6. Feasibility Outcomes

The feasibility outcomes were investigated in terms of acceptability (to what extent are the interventions suitable and satisfying for participants), implementation (to what extent can the interventions be successfully delivered remotely to participants), practicality (to what extent can the interventions be performed by participants using the intended means and resources), and limited efficacy (what are the preliminary impacts of the programs on study variables) [40]. Table 1 describes the feasibility outcomes, data sources, and analysis performed to assess each outcome.

### 2.7. Statistical Analysis

The IBM Statistical Package for Social Sciences software, version 27.0, and the Microsoft Excel app were used for storage and data analysis. A sample size calculation was not performed as it is a feasibility study. Therefore, we focused on determining the number of participants who could be recruited over six months in the two centers to estimate the time needed for sample recruitment in future trials. The Shapiro–Wilk test was used to test sociodemographic and clinical data distribution adopting a significance level of 5%. Continuous variables with normal distributions are expressed as the mean and standard deviation, while those with nonnormal distributions are expressed as the median and interquartile range. Categorical variables were summarized by absolute and/or relative frequencies (%). Additionally, the difference between post-and pre-intervention values (Δ post-pre) for study variables were calculated for analysis of the preliminary effects of the programs. The open answers to the satisfaction questionnaire and the participants’ questions received in the weekly follow-up were analyzed qualitatively (grouping by subject) and presented as text.

## 3. Results

**Figure 1:**
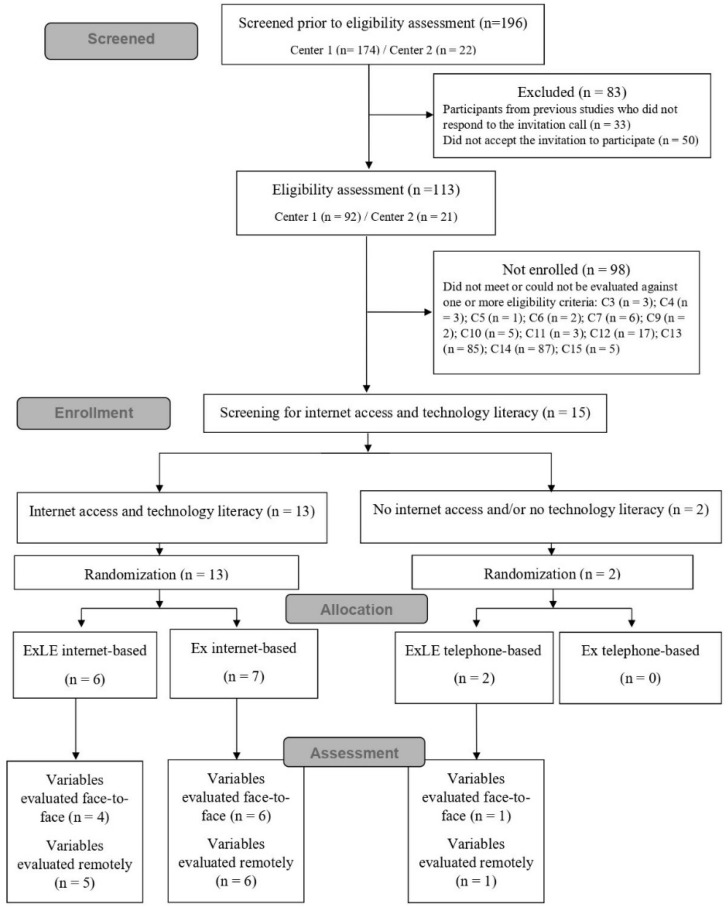
Flow diagram of participants through the study, including reasons for no eligibility assessment and no enrollment. C3: lack of cognitive limitation; C4: absence of unstable coronary artery disease; C5: absence of heart failure; C6: non-use of a pacemaker and/or implantable cardioverter defibrillator; C7: absence of intermittent claudication; C9:no complex arrhythmias; C10: no chest pain; C11: absence of a physical condition that limited or prevented the performance of exercises; C12: not currently participating in a structured exercise program; C13: written physician permission for exercising; C14: glycemic control; C15: literacy and without reading limitation; Ex: Exercise program; ExLE: Exercise and Lifestyle Education program.

Of 196 individuals screened, 113 were assessed for eligibility, and 15 met the eligibility criteria and agreed to be enrolled in the study. Only two participants had no internet access and/or no technology literacy, and both were randomized to the ExLE program, as illustrated in Figure 1. Therefore, there were no participants in the Ex telephone-based group.

Three participants, one from each group, dropped up during follow-up for reasons unrelated to the study. Of 12 participants that completed the program to which they were randomized and attended the remote stage of the post-intervention assessment, one (ExLE internet-based group) did not attend the face-to-face stage of the post-intervention assessment (Figure 1).

The sociodemographic and clinical characteristics of the participants in each group and the total sample are summarized in Table 2.

### 3.1. Acceptability

#### Satisfaction with the Interventions

Overall, most participants were satisfied or very satisfied with the exercise intervention (81.8%) and with the educational intervention (80%). However, some participants of the internet-based groups included difficulties related to website navigation and the registration of the interventions in the exercise and study diaries in response to the open questions of the satisfaction questionnaire, which are addressed in the subsequent section (Implementation).

### 3.2. Implementation

#### 3.2.1. Rate of Obtaining the Measures Used to Assess Study Variables

The completion rates in the pre-intervention assessment were 100% for all variables except for exercise self-efficacy and diabetes knowledge (93%). The completion rates in the post-intervention assessment were 100% for all variables measured in the remote stage. The completion rate of the variables measured in the face-to-face stage was as follows: functional capacity (91.3%), anthropometric assessment (91.3%), adherence to a Mediterranean food pattern (91.3%), physical activity level (83.3%), and HbA1c level (33.3%).

#### 3.2.2. Delivery Rate of the Filled Exercise and Study Diaries

The delivery rates of the filled aerobic exercise diaries, the filled muscle-strengthening exercise diaries, and the filled study diaries at the intervention completion were 71%, 28%, and 58%, respectively.

#### 3.2.3. Total Attempts and Answer Rate to the Phone Calls or Text Messages in the Weekly Follow-Up

Regarding the weekly follow-up of participants in the telephone-based group, 36 phone calls were made over the 12 weeks. The phone call attempts average was 1.46 per week per participant. The answering rate was 95%, considering the 2 participants allocated to this delivery format.

The analysis of the weekly follow-up data of participants in the internet-based groups revealed 232 WhatsApp text messages sent over the 12 weeks. The average number of WhatsApp text messages sent was 1.49 per week per participant. The response rate was 84.3%, considering the 13 participants allocated to this delivery format.

#### 3.2.4. Questions Asked by Participants during the Weekly Follow-Up

During the weekly follow-up, the research team received 24 questions from the participants that were answered by WhatsApp text messages or phone calls. The main topics of those questions were related to the ability/failure to reach the weekly study goals and how to achieve them (25%), the performance of aerobic exercises (21%), the performance of strengthening muscular exercises (17%), pulse taking (heart rate), interpretation of the pulse’s value (17%), orientations contained in the weekly exercise plans (8%), exercise diary filling (8%), and blood pressure response to the aerobic exercise (4%).

#### 3.2.5. Reported utilization rate of educational materials

All participants randomized for the ExLE program who attended the face-to-face stage post-intervention evaluation to deliver the study diaries back were considered in this analysis, regardless of the remote delivery format of the interventions (internet-based (*n* = 4) or telephone-based (*n* = 1)). The use rate for the lesson plans, patient guide, and videos or their transcripts (complementary materials) were 38%, 40%, and 58%, respectively.

#### 3.2.6. Website Visits and Barriers to Website Navigation

From August 2021 to May 2022, 138 visits to the Ex internet-based group website were verified, representing an average rate of 19.7 accesses overall (12 weeks) per participant or 1.6 weekly accesses per participant. On the ExLE internet-based group website were verified 152 visits from August 2021 to March 2022. The average access rate was 25 accesses overall (12 weeks) per participant, representing 2.1 weekly accesses per participant.

Regarding the barriers to website navigation, 33% of Ex internet-based group participants and 50% of the ExLE internet-based group reported some difficulty in accessing due to lack of time and trouble using the computer and browsing the website, in addition to the perception that the amount of information presented was too large and organized in a non-intuitive way. The participants’ suggestions for improving the educational intervention were to reduce and reorganize the vast amount of information available on the websites and to develop a mobile app that could replace the websites and facilitate access.

### 3.3. Practicality

#### Adherence Rate to Study Interventions

The exercise diary data analysis of all study participants revealed an adherence rate of 48% to aerobic and 24% to muscle-strengthening exercises. On average, the study participants performed 140 ± 122 min/week of aerobic exercise and 0.7 ± 1.2 days/week of muscle-strengthening exercises throughout the intervention period. The study diary analysis revealed an adherence rate of 58% to educational interventions, and the participants reviewed educational content on average for 65 ± 47 min/week.

### 3.4. Limited Efficacy

#### 3.4.1. Intervention Preliminary Effects on Study Variables

The total sample randomized for the ExLE program was considered in this analysis, regardless of the remote delivery format of the interventions. Both programs were associated with positive Δ post-pre for functional capacity (ExLE = 18.0 ± 49.7 m; Ex = 50.0 ± 119.2 m) and two of the eight health domains’ QofL measured, physical function (ExLE = 2.5 (−5.0–25.0); Ex = 5.0 (−25.0–10.0); expressed as median (minimum value–maximum value)) and general health (ExLE = 2.5 (−5.0–20.0); Ex = 12.5 (−20.0–30.0); expressed as median (minimum value–maximum value)). 

#### 3.4.2. Adverse Effects of Exercise Intervention

Regarding the possible adverse effects of the exercise intervention, only one participant reported moderate low back pain in the intervention’s 4th, 5th, and 6th weeks.

## 4. Discussion

This study has shown it feasible to remotely deliver the ExLE and Ex programs in an internet-based format. In contrast, the feasibility of remote delivering these programs in a telephone-based format could not be confirmed since most study participants had access to the internet and presented digital literacy, not needing a telephone-based delivery. The participants’ satisfaction was high with both study interventions, no serious adverse effects were reported, and data collection procedures and remote weekly follow-up worked adequately. The functional capacity seems to be positively affected by both programs. 

The remote internet-based delivery of the interventions could be improved with some adjustments, such as (1) study eligibility criteria change; (2) better organization of the content available on the websites; (3) new ways for participants to record the interventions; and (4) an initial supervised exercise session on site including all participants, regardless of the program to which the participant is randomized (ExLE or Ex), to assure that exercises are adequately performed. 

The most common eligibility criteria that prevented individuals’ enrollment in the study were glycemic control (C14) and written physician permission to exercise (C13). Of 113 individuals screened for eligibility, 87 had HbA1c levels beyond glycemic control criteria or did not have an HbA1c test performed three months before the pre-intervention assessment. Additionally, 85 of 113 individuals screened for eligibility could not get an appointment scheduled at the health service to request written physician permission to exercise within the recruitment period. Therefore, we recommend abolishing the eligibility criterion of glycemic control in future trials, considering the following: (1) many patients with diabetes are unable to get an HbA1c test every three months for either personal or financial reasons, including limited access to this test in the public health system and the national recommendation that HbA1c tests should be performed four times per year preferably for those who are changing their current treatment or not achieving recommended glycemic goals [30]; and (2) patients with HbA1c levels >7% might still benefit from exercise, given the effects of its regular practice on glycemic control [41]. However, written physician permission for exercising should be maintained as an eligibility criterion once the national diabetes guideline recommends that cardiovascular risk should be evaluated before prescribing exercise for individuals with type 1 [42] and type 2 diabetes [43]. In addition, this eligibility criteria maintenance will assure safety and prevent adverse effects of exercise. 

Regarding the study websites, the number of visits registered was within the expected range since the average number of visits per participant indicated that they had at least one access per week. In addition, the method of accessing the materials could be individualized; i.e., the participants could enter only once and download all the materials or access them more than once per week. Although most participants praised and were satisfied with the websites, some reported difficulty accessing them, indicating the need for some adjustments to facilitate and encourage navigation. Better organization of the available content in a more intuitive manner seems necessary, such as separating the materials into 12 blocks, one for each week of the intervention.

Whether the interventions were delivered remotely in two formats (internet or telephone-based), all participants received exercise and study diaries printed for the weekly recording of exercise sessions and studying of educational content. The diaries were used to measure adherence to the interventions. The participants were instructed to record in their diaries all exercise sessions performed, and all educational content studied during the 12-week intervention. However, the adherence rates to interventions might have been underestimated since some participants returned blank diaries. In this case, it is impossible to know whether they did not exercise or did not follow the educational program or whether they did not record it. On the satisfaction questionnaire, some participants reported a large amount of information to be filled out as a difficulty in the study and suggested, as an alternative, creating an application for the interventions, with which they could record all the information digitally. This idea seems promising, considering previous studies’ results [44,45]. Alternatively, participants could send text or audio messages to the research team to report the exercise sessions and educational content studied [46] or fill this information in electronic forms.

The weekly follow-up performed remotely via text message or phone worked properly, as demonstrated by the response rate greater than 80%. We observed that the participants generally reported using different educational materials; however, they did not entirely follow the study plan. Since the educational content of Diabetes College^TM^ was constructed through a rigorous process, informed by a theoretical foundation and adult learning principles [47,48], the information is repeated in the different materials, and participants can choose one or all of them according to their preferences and availability. The participants’ questions were relatively simple to answer remotely, and most were related to exercise. In this sense, in future trials, we recommend conducting an initial exercise session in person for all participants, regardless of the program (ExLE or Ex), with the opportunity to ask questions and ensure that everyone can experience an on-site exercise session.

The rates of obtaining the study variables in the pre- and post-intervention assessments were 100% for most variables, except for the DATE-Q and BESES questionnaires in the pre-intervention assessment and the measures obtained in the face-to-face stage of post-intervention evaluation. The data missing from the two self-administered questionnaires in the pre-intervention assessment was from a telephone-based group participant who was lost to follow-up. Consequently, this participant did not attend the post-intervention assessment to deliver back the questionnaires completed remotely in the pre-intervention assessment. Despite the high rates of obtaining the study variables, a possible strategy to prevent the missing data in the pre-intervention assessment would be to answer all questionnaires face-to-face.

Although it is not possible to conclude the effectiveness of the interventions, both programs seemed to promote beneficial changes in the functional capacity, which were verified by the increase in the average distance covered in the ISWT (positive Δ post-pre). The harmful effects of hyperglycemia on muscle strength and endurance and other factors, such as impaired glucose metabolism, chronic complications, and comorbidities, could contribute to reduced functional capacity in individuals with diabetes [3,49,50]. This reduction in functional capacity is related to poor prognosis due to increased cardiac risk and mortality [4,5]. Therefore, interventions that improve this outcome are clinically crucial for individuals living with diabetes and prediabetes.

This study has some limitations. The generalization of the results of the internet-based group is compromised since the number of participants enrolled in the study during the six months established for recruitment was low. In addition, the generalizability of the findings is limited as the participants were recruited from a single region in Brazil. However, these limitations do not prevent or limit the internet-based delivery of the programs.

## 5. Conclusions

The exercise and educational interventions delivered remotely using the internet appeared feasible and were well accepted by the participants. Although the findings indicate a potential clinical benefit of the programs on functional capacity, the actual effect will have to be tested by conducting a large-scale trial. Adjustments to eligibility criteria, study websites, ways of recording exercise sessions, using educational materials, as well as the execution of the first exercise session are recommended for future trials.

## Figures and Tables

**Table 1:** Feasibility outcomes, data sources, and data analysis.

	Feasibility Outcomes	Data Source	Data Analysis
Acceptability	- Participants’ satisfaction with the interventions	The satisfaction questionnaire answered by the participant in the face-to-face stage of post-intervention assessment	Descriptive statistics of numerical data and qualitative analysis of open questions of the satisfaction questionnaire
Implementation	- Rate of obtaining the measures used to assess study variables - The delivery rate of filled exercise and study diaries at the end of the interventions - Number of phone call attempts - Phone call response rate - Number of WhatsApp text messages sent - WhatsApp text messages response rate - Number and subject of questions received during the weekly follow-up - Reporting rate of use of each educational material - Number of visits to websites (assess rate) - Barriers to website navigation	Research files: assessment sheetsResearch files: exercise and study diaries Research files: spreadsheet used to report weekly follow-ups carried out by the research team Specific questions from the satisfaction questionnaire answered by the participant in the face-to-face stage of post-intervention assessment and study diary reports Data recorded on the website platform	Descriptive statistics of numerical data and qualitative analysis of the questions asked by the participants during the weekly follow-up and of the open questions of the satisfaction questionnaire
Practicality	- Adherence rate to exercise * and educational ** interventions	Research files: exercise and study diaries	Descriptive statistics of numerical data
Limited Efficacy	- Preliminary effects of the interventions on study variables (Δ post-pre) - Adverse effects of exercise intervention	Research files: data obtained from pre- and post-intervention assessments sheets and exercise diary	Descriptive statistics of numerical data

* Participants who performed ≥150 min per week of aerobic exercise were considered adherent to aerobic exercise, and participants who completed muscle-strengthening exercises twice or more per week were considered adherent to muscle-strengthening exercises, as recorded in the weekly exercise diaries. ** Participants who reported dedicating sometime a week to studies were considered adherent to the educational intervention, as recorded in the weekly study diaries. Δ post-pre: difference between post-and pre-intervention assessments.

**Table 2:** Sociodemographic and clinical characteristics of the participants.

Variables	ExLE Internet-Based (*n* = 6)	ExLE Telephone-Based (*n* = 2)	Ex Internet-Based (*n* = 7)	Total Sample (*n* = 15)
Sociodemographic Characteristics				
Age (years)	50.2 ± 11.3	70.5 ± 6.4	43.0 ± 14.9	49.5 ± 15.1
Female—% (*n*)	50.0 (3)	100.0 (2)	85.7 (6)	73.3 (11)
Years of study	16.7 ± 3.7	4.5 ± 0.7	16.7 ± 6.7	15.1 ± 6.5
Household income (USD)	2295.0 ± 2076.2 1735.8 (742.5–4050.1)	771.4 ± 0 771.4 (771.4–771.4)	2093.9 ± 1143.3 2507.2 (964.3–2893.0)	1998.0 ± 1535.3 1542.9 (771.4–2893.0)
Work status—% (*n*)				
Retired	16.7 (1)	100.0 (2)	14.3 (1)	26.7 (4)
Employed	83.3 (5)	0 (0)	57.1 (4)	60.0 (9)
Student	0 (0)	0 (0)	28.6 (2)	13.3 (2)
Marital status—% (*n*)				
Single	16.7 (1)	0 (0)	14.3 (1)	13.3 (2)
Married	83.3 (5)	50.0 (1)	71.4 (5)	73.3 (11)
Widow	0 (0)	0 (0)	14.3 (1)	6.7 (1)
Divorced	0 (0)	50.0 (1)	0 (0)	6.7 (1)
Clinical Characteristics				
Diabetes type—% (*n*)				
Type 1	16.7 (1)	-	0 (0)	6.7 (1)
Type 2	33.3 (2)	100.0 (2)	57.1(4)	53.3 (8)
Prediabetes	50.0 (3)	-	42.9 (3)	40.0 (6)
Time elapsed since diagnosis (years)	7.9 ± 8.8	3.2 ± 3.9	2.3 ± 2.4	2.0 (0.1–23.6)
Oral antidiabetics—% Yes (*n*)	66.7 (4)	100.0 (2)	85.7 (6)	80.0 (12)
Insulin therapy—% Yes (*n*)	16.7 (1)	0 (0)	0 (0)	6.7 (1)
Diabetes complications self-reported—% Yes (*n*)	0 (0)	50.0 (1)	0 (0)	6.7 (1)
Regular exercise self-reported—% Yes (*n*)	50.0 (3)	0 (0)	28.6 (2)	33.3 (5)
Smoking—% Yes (*n*)	16.7 (1)	0 (0)	0 (0)	6.7 (1)
Hypertension—% Yes (*n*)	33.3 (2)	100.0 (2)	14.3 (1)	33.3 (5)
Stress—% Yes (*n*)	16.7 (1)	100.0 (2)	42.9 (3)	40.0 (6)
Dyslipidemia—% Yes (*n*)	66.7 (4)	50.0 (1)	57.1 (4)	60.0 (9)
Healthcare service—% (*n*)				
Public	16.7 (1)	0 (0)	28.6 (2)	20.0 (3)
Private	66.7 (4)	50.0 (1)	57.1 (4)	60.0 (9)
Not reported	16.7 (1)	50.0 (1)	14.3 (1)	20.0 (3)

Values are expressed as mean ± standard deviation, median (interquartile range), or percentage (*n*). Ex: Exercise program; ExLE: Exercise and Lifestyle Education program.

## Data Availability

Not applicable.
